# Characterization of Pediatric Acute Lymphoblastic Leukemia Survival Patterns by Age at Diagnosis

**DOI:** 10.1155/2014/865979

**Published:** 2014-09-17

**Authors:** Md Jobayer Hossain, Li Xie, Suzanne M. McCahan

**Affiliations:** ^1^Nemours Biomedical Research, A. I. duPont Hospital for Children, 1600 Rockland Road, Wilmington, DE 19803, USA; ^2^The Department of Applied Economics and Statistics, University of Delaware, Newark, DE 19716, USA

## Abstract

Age at diagnosis is a key prognostic factor in pediatric acute lymphoblastic leukemia (ALL) survivorship. However, literature providing adequate assessment of the survival variability by age at diagnosis is scarce. The aim of this study is to assess the impact of this prognostic factor in pediatric ALL survival. We estimated incidence rate of mortality, 5-year survival rate, Kaplan-Meier survival function, and hazard ratio using the Surveillance Epidemiology and End Results (SEER) data during 1973–2009. There was significant variability in pediatric ALL survival by age at diagnosis. Survival peaked among children diagnosed at 1–4 years and steadily declined among those diagnosed at older ages. Infants (<1 year) had the lowest survivorship. In a multivariable Cox proportional hazard model stratified by year of diagnosis, those diagnosed in age groups 1–4, 5–9, 10–14, and 15–19 years were 82%, 75%, 57%, and 32% less likely to die compared to children diagnosed in infancy, respectively. Age at diagnosis remained to be a crucial determinant of the survival variability of pediatric ALL patients, after adjusting for sex, race, radiation therapy, primary tumor sites, immunophenotype, and year of diagnosis. Further research is warranted to disentangle the effects of age-dependent biological and environmental processes on this association.

## 1. Introduction

Leukemia is the most common childhood cancer worldwide [1]. Among the major types of leukemia, acute lymphoblastic leukemia (ALL) contributes to 76% of all leukemia cases and 43% of all deaths of pediatric leukemia patients in the US [2]. ALL is a malignant blood disorder that originates either from the T- or B-cell lineage, and it is hallmarked by subtype heterogeneity in chromosomal abnormalities, immunophenotypes, and treatment responsiveness [3]. Age at diagnosis has been recognized as an important prognostic factor of both incidence and survival of pediatric ALL, and it is incorporated into the NCI risk group classification [4–9]. The lowest survival is observed among patients diagnosed during infancy, followed by children who are diagnosed between 15 and 19 years of age [2, 5, 7, 8]. ALL patients diagnosed between 1 and 9 years of age have the highest chance of survival [2, 5, 7, 8]. Various studies have attempted to pinpoint specific genetic and biological processes occurring in different age groups to account for the prognostic value of age at diagnosis. For instance, cytogenetic markers such as the chromosomal rearrangement TEL/AML1 and DNA index ≥ 1.16, which peak at toddler and preschool age, are reported to be associated with favorable survival outcomes, while the BCR/ABL rearrangement, which is significantly lower in patients aged 1–4 years, are associated with unfavorable survival outcomes [10]. Similarly, older children have a higher percentage of T-cell immunophenotype, CD10-negative pB-ALL, BCR/ABL rearrangement, and a lower proportion of favorable biological features such as TEL/AML1 fusion and hyperploidy (DNA index ≥ 1.16) [10–15]. Other factors that influence the survival of pediatric ALL include race, sex, treatment, and primary cancer sites [16–27]. Studies have shown that females and non-Hispanic Caucasians have more favorable survival outcomes compared to others [24–27].

Though, in previous studies, the relationship between age at diagnosis and ALL survival has been consistently reported, limited studies have assessed the extent of this association after accounting for the effects of other known prognostic factors and taking the changing environment and ALL disease patterns into consideration. Published data are not only inadequate to explain the reason behind this association, but also inadequate in complete characterization of the extent of the survival variance of pediatric ALL over the age of diagnosis. Further research is warranted to quantify the effect of this important prognostic factor on pediatric ALL survival.

In this study, we have characterized the extent of the association of age at diagnosis and the survival patterns of pediatric ALL patients in the US, using the Surveillance Epidemiology and End Result (SEER) dataset between 1973 and 2009. The analysis has been controlled for sex, race, primary tumor sites, ALL immunophenotype, and utilization of radiation therapy. The SEER Program of the National Cancer Institute (NCI) is the most comprehensive and reliable source of population-based information in the United States on cancer incidence and survival [16]. The SEER dataset is large and well representative of ALL patients in the US, which is ideal for an epidemiological investigation like ours. Since the patients were diagnosed during 1973–2009, those diagnosed later had shorter follow-up period; that is, patients diagnosed in 1973 have as long as 36 years of follow-up time, while only 1 year or less follow-up time was for patients diagnosed in 2009. The risk of mortality has greatly reduced since 1973 to 2009 as the result of improved diagnosis and treatment [27–31]. The five-year survival rate was 43.2% in 1975 and 87% in 2005 [1–3]. In addition, there were changing risk factors, namely, lifestyle, diet, and maternal factors as well as clinical practice patterns such as early diagnosis and improved treatment protocol. In short, the survival patterns of pediatric ALL patients in the SEER dataset are not constant over the follow-up period. To account for this time-varying survival pattern, we stratified the analysis by the year of the diagnosis grouped into 5-year intervals.

## 2. Methods

The study included 14192 children who were diagnosed with ALL between ages 0 and 19 years during 1973–2009, whose information was reported to one of the 17 SEER registries. The SEER Program collects cancer data from 20 US geographic areas. These areas cover about 28% of the US population and are representative of the demographics of the entire United States population. In addition, the population covered by SEER is comparable to the general US population with regard to measures of poverty and education [2, 28]. The SEER registries collect population data on age, sex, race, year of diagnosis, primary tumor site, tumor morphology, and follow-up for vital status [2, 32]. The following study variables were included in the study.

### 2.1. Age at Diagnosis

As mentioned above, the study included ALL patients 0–19 years of age at the time of diagnosis. The SEER data included a variable of age at diagnosis recoded as <1, 1–4, 5–9, 10–14, and 15–19 years. This age classification is representative of age based pediatric ALL risk groupings used in most studies and was adopted for the purpose of this paper.

### 2.2. Year of Diagnosis

The study covered ALL patients diagnosed between 1973 and 2009. This variable was recorded in single-year interval. We recoded this variable into five-year intervals (1973–1977, 1978–1982, 1983–1987, 1988–1992, 1993–1997, 1998–2002, 2003–2007, and 2008-2009). The interval 2008-2009 contains only two years. The maximum follow-up period of this study is 37 years.

### 2.3. Sex

Sex was a nominal variable in the SEER dataset and used as a binary variable with female as the reference group.

### 2.4. Race

In the SEER dataset, the variable race contains information of White, Black, Asian/Pacific Islander, and others or unknown. Because of the limited number of subjects, we did not attempt to include the latter two categories in the analysis. We regrouped this variable as Caucasian (White), African American (Black), and others/unknown (Asian/Pacific Islander, others, or unknowns). African American (AA) was set as the reference group in our analyses.

### 2.5. Number of Primary Tumor Sites

The minimum number of primary tumor site was 1 in the SEER data, while the maximum number was 3. In our preliminary exploration, we found 1.1% of patients with 2 primary sites and 0.1% with 3 primary tumor sites. Hence, we collapsed the variable into two categories: one primary and two or more primaries. Patients with one primary tumor site were selected as the reference group.

### 2.6. ALL Immunophenotype

The information on the ALL immunologic features was available in the SEER dataset. We used two distinct immunophenotypes: T-cell and B-cell/B-precursor. This variable was treated as binary, with the T-cell as the reference group.

### 2.7. Radiation Therapy

Information of radiation therapy was listed in the SEER dataset as (a) beam radiation, (b) combination of beam radiation with implant or isotopes, (c) none, (d) radiation, not otherwise specified, (e) recommended, (f) refused, and (g) unknown. The variable was dichotomized into “yes, had radiation therapy of any kind” versus “no, never had radiation therapy during the time of data collection.”

### 2.8. Follow-Up Time and Survival Status

The follow-up time was documented as the duration from the time of diagnosis to death from any cause or the last day of the available survival information in the SEER registry. In the dataset, those who did not experience the event (death) during the follow-up time were censored. The survival status was determined on a binary scale, with 0 for censored and 1 for the event or failure.

### 2.9. Statistical Analyses

The overall and cancer-specific incidence rates of mortality (per thousand-person-month) were estimated by age at diagnosis. Clinical features of ALL were summarized by age group of diagnosis. Categorical variables were described using frequencies and percentages, while continuous variables were summarized using medians, 75% percentiles, means, and standard errors. Pearson's *χ* ^2^ statistic was used to examine the distribution of clinical features of ALL over age groups at ALL diagnosis, while the *χ* ^2^ trend test in proportion was performed to examine the distributional patterns of study variables over the age group at diagnosis. Five-year and ten-year survival rates were calculated by the age group at diagnosis. A univariable Cox proportional hazard model was performed to assess the effect of individual study variables including age group at diagnosis on survival of ALL. We utilized a multivariable Cox proportional hazard model, stratified by the year of diagnosis, to assess the extent of the association of age at diagnosis with the survival of ALL after accounting for the effect of other influential factors found in the univariable model. In this regard, we performed two adjusted models with and without the inclusion of immunophenotype in the model. The variable immunophenotype had 5881 missing values. For both adjusted models, stratified analyses were performed by the year of diagnosis to account for the time varying survival in the follow-up period. The statistical software R version 2.15.1 (R Foundation for Statistical Computing, Vienna, Austria) and STATA 10.0 (STATACorp, College Station, TX) were used to perform the analyses.

## 3. Results

Of the 14192 ALL patients, the overall rate of diagnosis increased with age at diagnosis before the age of three years and decreased afterwards, peaking at the age of two. Forty-six percent of the total patients were diagnosed between ages 1 and 4 years. Interestingly, the age group (1–4 years) in which most ALL diagnoses were made was also the group that experienced the lowest mortality rate.  Table 1(a) presented the all-cause and cancer-specific incidence rates of dying per thousand-person-month. Children who were diagnosed in infancy had the highest mortality rate, whereas those diagnosed at the age of 3 years had the lowest mortality rate. Both overall and cancer-specific mortality rates steadily increased as age at diagnosis increased in children older than 3 years.

Table 1(b) displayed the characterization of demographics and other study variables by age group at diagnosis. Compared to females, more male children were diagnosed except in the infant group. There was a steadily increasing trend of the proportion of male children as the age at diagnosis increased (*χ* ^2^ for trend = 79.43, df = 1, and *P* < 0.0001). There was a marginal difference in the distribution of race over the age at diagnosis groups (*χ* ^2^ = 54.92, df = 8, and *P* = 0.0551). The majority (83%) of diagnosed children were Caucasian. The proportion of AA was about 7%. The proportion of children having multiple primary tumor sites at the time of diagnosis monotonically increased with age at diagnosis (*χ* ^2^ for trend = 5.74, df = 1, and *P* = 0.017). This proportion was 1% among children diagnosed during infancy and 2% for those diagnosed between 15 and 19 years of age. The proportion of children with T-cell ALL steadily increased as the age at diagnosis increased (*χ* ^2^ for trend = 302.77, df = 1, and *P* < 0.0001). Whereas, during infancy, only 3% of ALL diagnoses were of the T-cell immunophenotype, the proportion increased to 14% in children diagnosed between 15 and 19 years.

Radiation therapy utilization significantly increased with increased age at diagnosis (*χ* ^2^ for trend = 361.10, df = 1, and  *P* < 0.0001). Beginning with the 10–14 years of age group, there was a sharp rise in the proportion of pediatric ALL patients undergoing radiation therapy. Among those who were diagnosed between 5 and 9 years, 17% underwent radiation therapy, while this proportion was 29% among children diagnosed 10–14 years. There was a marginal difference in the distribution of pediatric ALL patients by age at diagnosis over the year of diagnosis (*χ* ^2^ = 44.05, df = 28, and *P* = 0.027). The number of diagnosis increased over the years partly due to the expansion of the SEER program.

Seventy-five percent of ALL patients diagnosed between ages 5 and 9 years survived at least 164 months after diagnosis, while the same proportion of children in age-at-diagnosis groups <1, 10–14, and 15–19 years survived at least 11, 44, and 22 months, respectively. This statistic could be not calculated for children diagnosed between 1 and 4 years of age, because more than 25% of children did not experience mortality. Median survival time results could be interpreted in the same fashion. Children diagnosed with ALL during infancy had the lowest 5-year interval survival rates. Children diagnosed between ages 1 and 4 years represented the highest proportion of those who survived each 5-year period, followed by the 5–9, 10–14, and 15–19 years of age at diagnosis groups. The 5-year survival rate was 46%, 87%, 83%, 71%, and 57% among children diagnosed at <1, 1–4, 5–9, 10–14, and 15–19 years of age, respectively. A significant difference was observed in the survival status with respect to the age group at diagnosis (*χ* ^2^ = 871.65, df = 4, and *P* < 0.0001). The highest proportion of overall survival (86.0%) occurred in the 1–4 years age at diagnosis group and decreased among those who were diagnosed with ALL at later ages.

Table 2 showed the association of pediatric ALL survival with the age at diagnosis as well as other known prognostic factors available in the SEER dataset. Compared to the reference group (infants) the estimated hazard ratio (95% confidence interval (CI)) of children diagnosed with ALL in the 1–4, 5–9, 10–14, and 15–19 age groups were 0.18 (0.15–0.20), 0.26 (0.2–0.30), 0.44 (0.38–0.51), and 0.69 (0.59–0.80), respectively. In other words, children diagnosed with ALL between ages 1 and 4 years, 5 and 9 years, 10 and 14 years, and 15 and 19 years were 82%, 75%, 56%, and 30% less likely to die compared to those diagnosed at infancy. The above results indicated that the children diagnosed between ages 1 and 4 years had the lowest risk of mortality and that though the risk of mortality continuously increased among children diagnosed at older ages, it never exceeded the risk of mortality experienced by those diagnosed with ALL during infancy. Compared to females, male children diagnosed with ALL on average were expected to have 1.29 times higher hazard of death (HR (95% CI) = 1.29 (1.20–1.39)). AA showed a significantly higher risk of mortality compared to that of Caucasian (HR (95% CI) = 1.44 (1.27–1.62)). Although not significant, children diagnosed with multiple primary tumor sites showed a higher risk of death compared to those who were with a single primary tumor site (HR (95% CI) = 1.18 (0.92–1.52)). Children with the B-cell and B-precursor ALL had a lower hazard than those with T-cell ALL (HR (95% CI) = 0.57 (0.49–0.65)). Compared to the children who did not undergo radiation therapy, the irradiated children had an approximate 1.66 times higher hazard (HR (95% CI) = 1.66 (1.53–1.79)). The HR of ALL mortality decreased monotonically over the year of diagnosis, which implied the time-varying survival pattern during the follow-up period.

Table 3 presented the association of the pediatric ALL survival with the age at diagnosis after accounting for the effects of the influential covariates mentioned above using a multivariable Cox proportional hazard model, stratified by the year of diagnosis. Specifically, the left side of Table 3 presented the estimates from a multivariable model that accounted for the effects of sex, race, number of primary tumor sites, and radiation, while the right side presented the results from a model that included all factors examined in the aforementioned model and immunophenotype. After accounting for the effects of other covariates and stratification by the year of diagnosis, the age at diagnosis remained to be a highly significant factor associated with the survival of diagnosed pediatric ALL patients. Compared to children diagnosed during infancy, children whose ages at diagnosis were 1–4 years, 5–9 years, 10–14 years, and 15–19 years were 82% (AHR (95% CI) = 0.18 (0.15–0.20)), 75% (AHR (95% CI) = 0.25 (0.22–0.29)), 57% (AHR (95% CI) = 0.43 (0.37–0.50)), and 32% (AHR (95% CI) = 0.68 (0.59–0.79)) less likely to die, respectively. The rise in the adjusted hazard ratio (AHR) over the year of diagnosis revealed that after infancy, children who were older at the time of diagnosis were at an increasingly higher risk of mortality. Besides the effect of age at diagnosis, the impact of sex, and race on pediatric ALL survivorship persisted in the results of the multivariable model. Similarly, after further adjustment for the ALL immunophenotype, the relationship between age at diagnosis and pediatric ALL survival still persisted. We used the same models for the analysis of ALL specific mortality instead of overall mortality. The difference in the results of the two sets of models was negligible. Hence, the results using the overall mortality status were presented.

**Figure 1:**
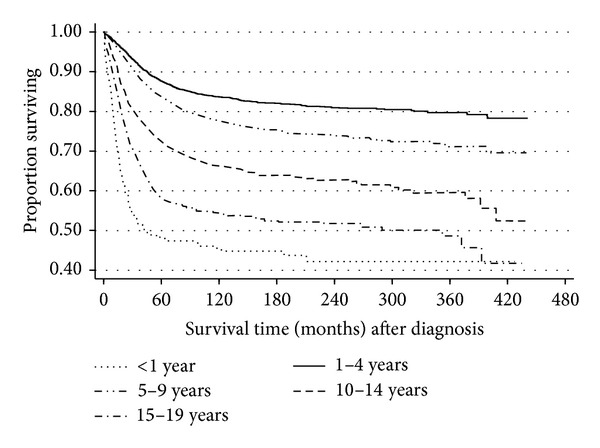
Kaplan-Meier pediatric ALL survival estimates by age at diagnosis.

Figure 1 showed the Kaplan-Meier survival estimates of the distinct survival patterns among pediatric ALL patients by age at diagnosis group. Children diagnosed between ages 1 and 4 years had the highest probability of survival during the follow-up period, followed by those diagnosed between ages 5 and 9 years, while children diagnosed during infancy had the lowest survival pattern. Figure 1 displayed a sharp decline in survival probability of pediatric ALL during the first five years after diagnosis. Thereafter, the declines in the curves became gradual.

## 4. Discussion

The prognostic value of age at diagnosis in pediatric ALL has long been recognized, but this variable has not been adequately assessed in terms of the extent of its effect on survival. The present study is conducted to assess the effect of age at diagnosis on the survival patterns of children diagnosed with ALL. Our main finding is that there is a significant variation in survival by age at diagnosis, with the worst outcome for children diagnosed in infancy, the best outcome for those diagnosed during the age of 1–4 years, and a monotonically decreasing trend in survival for those diagnosed after 4 years. The differential survival patterns of pediatric ALL by age at diagnosis persist after accounting for the effects of known prognostic factors: sex, race, receipt of radiation therapy, ALL immunophenotype, and the number of primary tumor sites. These patterns may be partly due to a variety of age-dependent favorable and unfavorable clinical and biological features mentioned in the introduction.

The previous studies have identified sex [25, 33–36], race [24, 32], number of primary tumor sites [33], radiation therapy [37], chemotherapy [38] and immunophenotype [16–27] as important predictors of ALL survival. Our study has confirmed all of these results of previous studies except chemotherapy, which is not available in the SEER data.

In the SEER dataset, we have observed increased proportion of boys, children with multiple primary tumor sites, T-cell ALL phenotype, and radiation therapy recipients as age at diagnosis increased. These factors are associated with increased risk of mortality (Table 2), which may partially explain the declining survival trend with an increased age at diagnosis after 4 years.

As discussed in the Introduction, ALL survival has improved dramatically among children due to the improved treatment and early diagnosis over the years, causing the baseline hazard to differ within the study period. The stratified analysis technique has been used to overcome this constraint.

Like other epidemiological studies, the current study is not without limitation. First, our results may be driven in part by the effect of unmeasured confounders. There is limited information of treatment (radiation therapy) in the SEER dataset. Secondly, the follow-up periods tend to be shorter for children diagnosed more recently. However, our results are not limited by this variability of the follow-up time, since we have stratified the analysis by the year of diagnosis.

In summary, there is a differential survival pattern of pediatric ALL by age at diagnosis. Unique biological and environmental processes occurring at different stages of development may give rise to this association. Future research could focus on identifying these processes and elucidating their mechanisms.

## Figures and Tables

**(a):** 

Age at diagnosis (yr)	Number of patients (%)	Number of deaths (%)	All-cause incidence rate of dying per 1000 person-month	Cancer-specific incidence rate of dying per 1000 person-month
0	452 (3)	229 (51)	8.35	7.47
1	984 (7)	206 (21)	2.07	1.76
2	1968 (14)	250 (13)	1.13	0.93
3	1936 (14)	231 (12)	1.06	0.88
4	1495 (11)	205 (14)	1.28	1.07
5	1025 (7)	168 (16)	1.48	1.23
6	771 (5)	138 (18)	1.74	1.54
7	665 (5)	134 (20)	1.97	1.73
8	541 (4)	108 (20)	1.87	1.63
9	483 (3)	124 (26)	2.84	2.54
10	437 (3)	119 (27)	2.86	2.45
11	444 (3)	120 (27)	3.26	2.80
12	413 (3)	121 (29)	3.29	2.75
13	436 (3)	146 (33)	4.07	3.62
14	445 (3)	140 (31)	3.96	3.17
15	419 (3)	167 (40)	5.44	4.63
16	395 (3)	120 (30)	3.87	3.42
17	321 (2)	129 (40)	5.98	5.38
18	309 (2)	150 (48)	8.19	7.43
19	253 (2)	115 (45)	7.70	7.10

**(b):** 

Variables	Age group at diagnosis (yrs)						
	<1 (%)	1–4 (%)	5–9 (%)	10–14 (%)	15–19 (%)	*χ* ^2^	*P*
Sex						110.14	<0.0001
Male	217 (48)	3553 (56)	1917 (55)	1273 (59)	1153 (68)		
Female	235 (52)	2830 (44)	1568 (45)	902 (42)	544 (32)		
Race						54.92	0.05
Caucasian	360 (80)	5353 (84)	2876 (83)	1793 (82)	1435 (85)		
AA	44 (10)	373 (6)	255 (7)	208 (10)	125 (7)		
Other/unknown	48 (11)	657 (10)	354 (10)	174 (8)	137 (8)		
Primary tumor sites						5.85	0.21
1	448 (99)	6313 (99)	3437 (99)	2142 (98)	1668 (98)		
≥2	4 (1)	70 (1)	48 (1)	33 (2)	29 (2)		
Immunophenotype						375.77	<0.0001
T-cell	13 (3)	223 (3)	376 (11)	306 (14)	233 (14)		
B-cell, B-precursor	217 (48)	3389 (53)	1698 (49)	1005 (46)	851 (50)		
Radiation						419.28	<0.0001
Yes	61 (14)	785 (12)	574 (17)	616 (29)	462 (27)		
No	388 (86)	5569 (88)	2895 (83)	1537 (71)	1219 (73)		
Year of diagnosis						44.05	0.027
1973–1977	22 (3)	328 (5)	187 (5)	122 (6)	93 (5)		
1978–1982	33 (4)	371 (6)	202 (6)	130 (6)	106 (6)		
1983–1987	35 (4)	430 (7)	221 (6)	145 (7)	107 (6)		
1988–1992	42 (4)	552 (9)	303 (9)	151 (7)	110 (6)		
1993–1997	53 (3)	852 (13)	438 (13)	244 (11)	172 (10)		
1998–2002	99 (3)	1357 (21)	782 (22)	476 (22)	381 (22)		
2003–2007	126 (3)	1790 (28)	943 (27)	638 (29)	522 (31)		
2008-2009	42 (3)	703 (11)	409 (12)	269 (12)	206 (12)		
Survival time (months)							
75th percentile	11	—	164	44	22		
Median	40	—	—	—	289		
Mean (SE)	198 (11.26)	364 (2.47)	335 (3.80)	282 (5.51)	233 (6.55)		
Five-year interval Survival (months)							
0–60	46	87	83	71	57		
61–120	43	83	77	65	53		
Mortality status							
Dead	229 (51)	892 (14)	672 (19)	646 (30)	681 (40)	871.65	<0.0001
Alive	223 (49)	5491 (86)	2813 (81)	1529 (70)	1016 (60)		

**Table 2:** Hazard risk of mortality associated with age at diagnosis in pediatric ALL patients (1973–2009 SEER dataset) in an univariable Cox proportional hazard model.

Variables	Hazard ratio (HR)	95% C.I.*	*P*
Age at diagnosis (yrs)			
<1	1.00	—	Reference
1–4	0.18	0.15, 0.20	<0.0001
5–9	0.26	0.22, 0.30	<0.0001
10–14	0.44	0.38, 0.51	<0.0001
15–19	0.69	0.59, 0.80	<0.0001
Sex			
Female	1.00	—	Reference
Male	1.29	1.20, 1.39	<0.0001
Race			
Caucasian	1.00	—	Reference
AA	1.44	1.27, 1.62	<0.0001
Primary tumor sites			
1	1.00	—	Reference
≥2	1.18	0.92, 1.52	0.1950
Immunophenotype			
T-cell	1.00	—	Reference
B-cell, B-precursor	0.57	0.49, 0.65	<0.0001
Radiation			
No	1.00	—	Reference
Yes	1.66	1.53, 1.79	<0.0001
Year of diagnosis			
1973–1977	1.00	—	Reference
1978–1982	0.70	0.61, 0.80	<0.0001
1983–1987	0.56	0.49, 0.65	<0.0001
1988–1992	0.38	0.33, 0.44	<0.0001
1993–1997	0.32	0.28, 0.36	<0.0001
1998–2002	0.29	0.26, 0.33	<0.0001
2003–2007	0.24	0.21, 0.27	<0.0001
2008-2009	0.19	0.14, 0.25	<0.0001

*C.I.: confidence interval.

**Table 3:** Hazard risk of mortality associated with age at diagnosis in pediatric ALL patients (1973–2009 SEER dataset) in an adjusted multivariable Cox proportional hazard model, stratified by the year of diagnosis.

Variable	Model without immunophenotype			Model with immunophenotype		
	Adjusted hazard ratio (AHR)	95% C.I.*	*P*	Adjusted hazard ratio (AHR)	95% C.I.	*P*
Age at diagnosis (yr)						
<1	1	—	Reference	1	—	Reference
1–4	0.18	0.15, 0.20	<0.0001	0.13	0.10, 0.16	<0.0001
5–9	0.25	0.22, 0.29	<0.0001	0.20	0.16, 0.26	<0.0001
10–14	0.43	0.37, 0.50	<0.0001	0.37	0.29, 0.46	<0.0001
15–19	0.68	0.59, 0.79	<0.0001	0.60	0.48, 0.75	<0.0001
Sex						
Female	1	—	Reference	1	—	Reference
Male	1.25	1.16, 1.34	<0.0001	1.23	1.09, 1.38	0.0004
Race						
Caucasian	1	—	Reference	1	—	Reference
AA	1.44	1.27, 1.62	<0.0001	1.33	1.10, 1.61	0.003
Primary tumor sites						
1	1	—	Reference	1	—	Reference
≥2	0.86	0.67, 1.11	0.25	1.45	0.94, 2.23	0.09
Radiation						
No	1	—	Reference	1	—	Reference
Yes	0.94	0.87, 1.03	0.15	1.02	0.89, 1.18	0.77
Immunophenotype						
T-cell	—	—	—	1	—	Reference
B-cell, B-precursor	—	—	—	0.84	0.73, 0.96	0.01

*C.I.: confidence interval.
